# The Content of Certain Groups of Phenolic Compounds and the Biological Activity of Extracts of Various Halophyte Parts of *Spergularia marina* (L.) Griseb. and *Glaux maritima* L. at Different Levels of Soil Salinization

**DOI:** 10.3390/plants11131738

**Published:** 2022-06-30

**Authors:** Artem Pungin, Lidia Lartseva, Violetta Loskutnikova, Vladislav Shakhov, Olesya Krol, Elena Popova, Andrey Kolomiets, Nadezhda Nikolaeva, Aleksandra Volodina

**Affiliations:** Institute of Living Systems, Immanuel Kant Baltic Federal University, Universitetskaya Str. 2, 236040 Kaliningrad, Russia; lida.lartseva@mail.ru (L.L.); violett.loskutnikova@gmail.com (V.L.); vladlyshakhov@gmail.com (V.S.); okrol@kantiana.ru (O.K.); elena_popova97@mail.ru (E.P.); kolomiez1997@gmail.com (A.K.); nanikolaeva1999@gmail.com (N.N.); volodina.alexandra@gmail.com (A.V.)

**Keywords:** halophytes, soil salinization, *Spergularia marina*, *Glaux maritima*, secondary metabolites, flavonoids, hydroxycinnamic acids, antioxidant activity, antibacterial activity, fungicidal activity

## Abstract

Halophyte plants are known for their resistance to harsh environmental conditions associated with excess salts in their habitats. Their resistance to salinization is due, among other things, to their high ability to detoxify free radicals, owing to the relatively high content of antioxidants. On the coast of the Baltic Sea and in the lagoons, there are several rare halophyte species included in the Red Book of the Kaliningrad Region (Russia) and the Baltic region, such as *Spergularia marina* (L.) Griseb. and *Glaux maritima* L. The aim of the research was to study the accumulation of certain groups of phenolic compounds in different parts of *S. marina* and *G. maritima* plants under conditions of weak and strong soil salinity, as well as to analyze the antioxidant, antibacterial, and fungicidal activity of extracts of the studied plant species. The present study showed an increase in total phenolic content in the roots and shoots of *S. marina,* and the shoots of *G. maritima,* in response to increased soil salinity. At the same time, the total content of flavonoids in all the studied parts of the two plant species remained unchanged. However, the content of individual flavonoids (hesperetin, epicatechin, apigenin derivative, luteolin derivative) in *S. marina* increased, for *G. maritima* there was a tendency to reduce the content of flavonoids in roots and shoots with an increase in soil salinity. There was an increase in the total content of hydroxycinnamic acids in the roots of *Glaux maritima*, as well as an increase in the content of protocatechuic acid in the roots and shoots of *Spergularia marina*. A positive relationship was established between the antioxidant activity of *S. marina* root extracts and the total content of phenolic compounds, as well as *G. maritima* shoots extracts and the total content of phenolic compounds. Extracts of *S. marina* showed no antibacterial activity against *Escherichia coli* and *Bacillus subtilis*, and weak fungicidal activity of stem extracts and inflorescences grown on soils, with high levels of salinities, was detected against *Candida albicans*. The extracts of roots and shoots from *G. maritima* showed weak antimicrobial and fungicidal activity.

## 1. Introduction

It is known that many of the natural biologically active substances used in medicine are products of the secondary metabolism of plants, which have various beneficial effects on human health [1]. The therapeutic effect of biologically active substances such as phenolic compounds is possible due to their antioxidant activity [2]. Antioxidants play a significant role in protecting the cell from reactive oxygen species and free radicals [3]. Excess free radicals in the cell cause oxidative stress, which leads to premature aging, the development of diabetes, cardiovascular and neurological diseases, and cancer [4,5]. Plants have effective antioxidant mechanisms that protect them from the toxic effect of free radicals, which are formed in large quantities in plant cells due to physiological and biochemical features [6].

Glycophyte plants are not adapted to salt stress, which leads to various adverse changes, including the excessive formation of free radicals [7,8]. Halophytes, by contrast, are known for their resistance to harsh environmental conditions associated with excess salts in their habitats [7,8]. Their resistance to such environmental conditions is due, among other things, to their high ability to detoxify free radicals, which is possible due to the relatively high content of antioxidants, including compounds of polyphenolic nature [9]. It has been shown that the total amount of polyphenols in halophytes is twice as high in glycophytes, and the extracts from halophytes have a high ability to remove free radicals of DPPH (2,2-diphenyl-1-picrylhydrazyl) and reduce the activity of Fe^3+^ compared to glycophytes, which may indicate the important role of polyphenols in reducing the harmful effects of free radicals in halophyte species [10,11].

On the coast of the Baltic Sea and in the lagoons, there are several rare halophyte species listed in the Red Book of the Kaliningrad Region [12] and the Baltic region [13]. Two rare halophyte species are of particular interest: *Spergularia marina* (L.) Griseb. and *Glaux maritima* L. From a global perspective, these plants is considered a least-concern species [14,15].

*S. marina*, an annual herbaceous plant of the Caryophyllaceae family, grows in Europe, North Africa, Asia, Australia, and North America [16]. As a mandatory halophyte, this species grows on soils with variable but usually high salinity. It is found in marine and inland salt marshes, and can inhabit roadsides on which salt has been used to melt ice [16].

*G. maritima* is a perennial herbaceous plant belonging to the Primulaceae family. This species is also known as *Lysimachia maritima* (L.) [17,18]. The species is circumpolar in the northern hemisphere and grows in Europe, Central Asia, and North America. It prefers to grow in very humid, salty places, which can also be flooded periodically, usually in coastal habitats [19,20].

The biological activity and chemical composition of secondary metabolites of *S. marina* remain poorly studied. In the study of several species of the genus *Spergularia*, the therapeutic effects and properties of extracts were revealed: hypoglycemic, diuretic, hypotensive, antibacterial, antinociceptive, anti-inflammatory, antioxidant, and cholesterol-lowering properties [21,22]. It is known that *S. marina* is used in the food industry in South Korea for the development of functional food products and as a supplement—a substitute for salt [23,24,25]. *S. marina* has been found to have such properties as reduced insulin resistance [26,27], antioxidant activity, anti-inflammatory [28,29,30], antimicrobial [31], and antiadipogenic effects [27], as well as the enhancement of osteoblast differentiation [28] and an inhibitory effect on cancer cells [32]. Vasodilatory and diuretic effects of extracts have been noted, which represents the prospect of *S. marina*-based preparations as calcium channel antagonists with antihypertensive effects [33].

The biological activity and chemical composition of secondary metabolites of *G. maritima* have not yet been determined [34,35]. Species of the *Lysimachia* genus are used in traditional medicine in East Asia to treat digestive disorders, stop bleeding, and sterilize wounds [36]. Extracts of these plants are known to contain polyphenols [37] and saponins [38]. It has been shown that extracts of species of the *Lysimachia* genus have antioxidant [39,40,41], anthelmintic [42], antimicrobial [43], and endothelioprotective effects [41], and they can improve the vasodilating function of the endothelium [44], inhibit lipogenesis processes [36], and exhibit therapeutic effects on neurodegenerative diseases [45].

As can be seen, the studied plants have a high biotechnological and therapeutic potential, but the chemical composition of secondary metabolites remains poorly studied. Moreover, it is not known how the growing conditions, in particular the level of soil salinization, affect the qualitative and quantitative composition of secondary metabolites. Based on this, the purpose of this work was to study the accumulation of certain groups of phenolic compounds in different parts of *Spergularia marina* and *Glaux maritima* plants under conditions of weak and strong soil salinity, as well as to analyze the antioxidant, antibacterial, and fungicidal activity of extracts of the studied plant species.

## 2. Results

### 2.1. Soil Analysis

A soil chemistry analysis was performed; the mean and standard deviation of pH, electrical conductivity, soluble salt residue, soluble cations, and anions in the soil are shown in Table 1. As a result of the analysis, it was found that the soil used for the studied plants was generally alkaline (pH = 7.56–7.74). The electrical conductivity, dry residue, chloride, sulfate, sodium, potassium, and calcium contents were high in the soil at the elevated areas of *S. marina* and *G. maritima* (upper level of the salt marsh), while in the soil in the relief depressions (lower level of the salt marsh), the values were significantly lower.

It is known that soil salinization gradually increases with height, reaching a maximum just above the average sea level [46,47]. This can be explained by the fact that the evaporation periods (occurring when the area is not flooded) are longer at higher altitudes than in lowlands, and thus the salts in the surface soils can be very concentrated. In higher ground areas, above mean sea level, soil salinity tends to decrease due to the increasingly rare solonchak flooding and the consequent decrease in salt intake.

According to the agronomic classification of soil salinization based on electrical conductivity [48], the soil on the upper level of *S. marina* and *G. maritima* can be characterized as saline soils, and in turn, the soil in the lower level is non-saline. However, a relatively high content of chlorides and sodium was found in the terrain downgrades, indicating the presence of salinization.

According to another classification, which is based on the assessment of the dry residue of salts readily soluble in water [49], the soil in the upper level is characterized as highly saline, and in the lower level as slightly saline. Further in this work, the authors will adhere to this classification when assessing the total content of phenolic compounds, flavonoids, and hydroxycinnamic acids, and when evaluating the antioxidant, antimicrobial, and fungicidal activity of extracts of *S. marina* and *G. maritima* at different levels of soil salinization.

### 2.2. Total Phenolic Compounds Content

**Figure 1:**
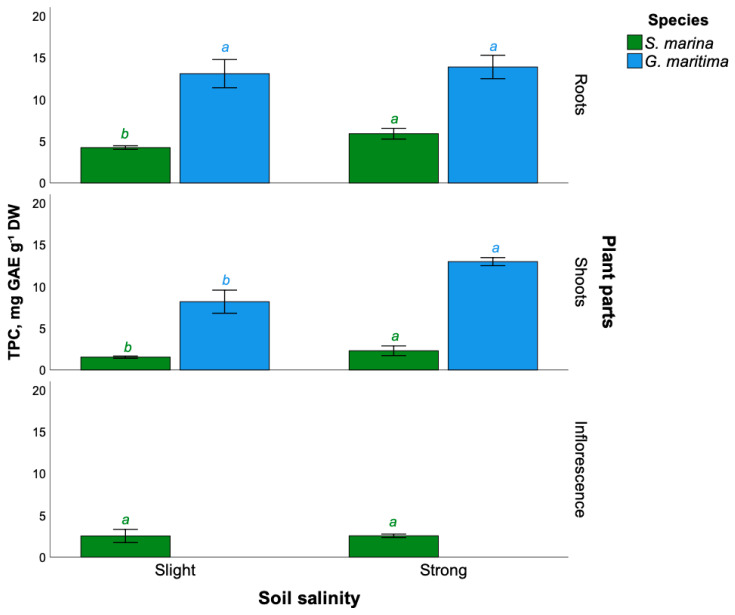
The total content of phenolic compounds in different parts of the studied plants of *S. marina* and *G. maritima* at different levels of soil salinization. Different letters indicate significant differences among the different levels of soil salinity (*t*-test, *p* ≤ 0.05).

The total content of phenolic compounds in different parts of the studied plants of *S. marina* and *G. maritima* at different levels of soil salinization was found (Figure 1). For *S. marina*, the highest content of phenolic compounds was found in plant roots (5.9 ± 0.6 mg GAE g^−1^ DW) growing on soils with a high level of salinity. Low total phenolic content (TPC) was observed in *S. marina* shoots (1.5 ± 0.1 mg GAE g^−1^ DW) under low salinity conditions. The total content of phenolic compounds in roots and shoots significantly increases with an increase in soil salinity.

In *G. maritima*, high total content of phenolic compounds was found in the roots (13.9 ± 1.4 mg GAE g^−1^ DW) under conditions of strong soil salinity, but there were no significant differences with such content under conditions of weak soil salinity (*t*-test, *p* > 0.05). However, differences in the total content of phenolic compounds in *G. maritima* shoots with an increase in soil salinity were found from 8.2 ± 1.4 mg GAE g^−1^ DW with a low level of salinity to 13.0 ± 0.5 mg GAE g^−1^ DW under conditions of strong soil salinity. 

### 2.3. Total Flavonoids Content

**Figure 2:**
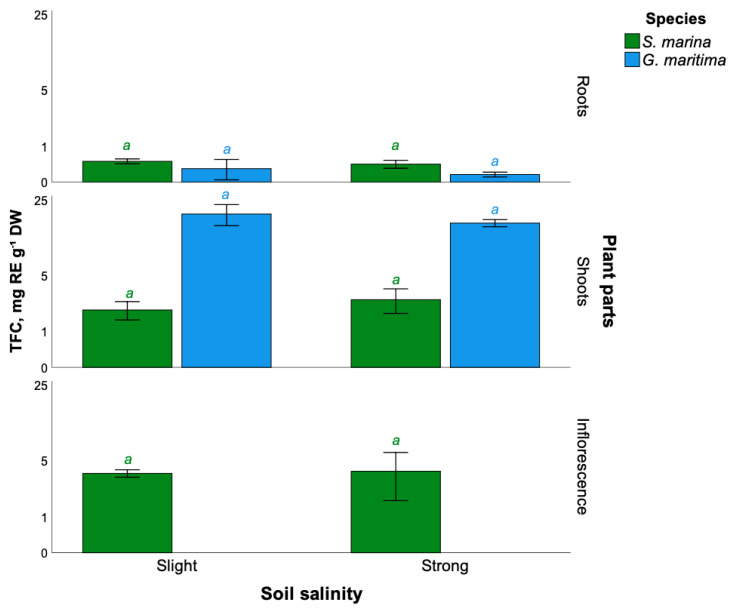
The total content of flavonoids in different parts of the studied plants of *S. marina* and *G. maritima* at different levels of soil salinization. Different letters indicate significant differences among the different levels of soil salinity (*t*-test, *p* ≤ 0.05).

The analysis of the total content of *S. marina* flavonoids showed that the maximum content of flavonoids was in inflorescences (3.9 ± 2.1 mg RE g^−1^ DW), and the minimum was in the roots (0.4 ± 0.1 mg RE g^−1^ DW). In *S. marina* shoots, under conditions of strong soil salinity, the total content of flavonoids was 2.7 ± 0.9 mg RE g^−1^ DW. However, the authors did not find differences in the content of flavonoids in the roots, shoots, and inflorescences of *S. marina* growing in conditions of weak and strong soil salinization (Figure 2).

In turn, for *G. maritima,* growing at different levels of soil salinization, no differences in the content of flavonoids in roots and shoots were found. Concurrently, the highest content of flavonoids was found in the shoots of plants under conditions of weak soil salinization (18.8 ± 4.0 mg RE g^−1^ DW), and the lowest was found in the roots of plants with strong soil salinization (0.2 ± 0.1 mg RE g^−1^ DW).

### 2.4. Total Hydroxycinnamic Acids Content

The content of hydroxycinnamic acids in various parts of *S. marina* could not be determined, due to the low content of these substances, which is below the sensitivity threshold of the method used.

**Figure 3:**
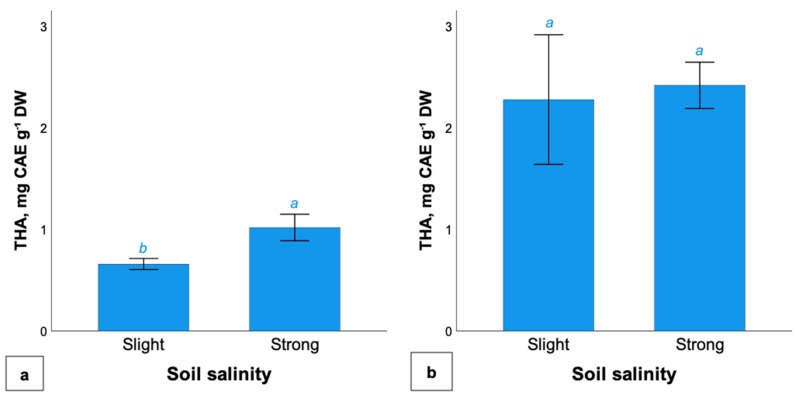
Total hydroxycinnamic acids in roots (**a**) and shoots (**b**) of *G. maritima* at different levels of soil salinization. Different letters indicate significant differences among the different levels of soil salinity (*t*-test, *p* ≤ 0.05).

The results of the study of the accumulation of hydroxycinnamic acids in various parts of *G. maritima* are presented in Figure 3. The analysis showed that the accumulation of hydroxycinnamic acids in *Glaux* roots depended on the level of soil salinity; a higher content was observed at a high level of salinity (1.0 ± 0.1 mg CAE g^−1^ DW). In the shoots of *G. maritima*, the content of hydroxycinnamic acids was more than twice as high as that of roots, while no dependence of the content on the level of soil salinity was observed.

### 2.5. Content of Individual Phenolic Compounds

**Figure A1:**
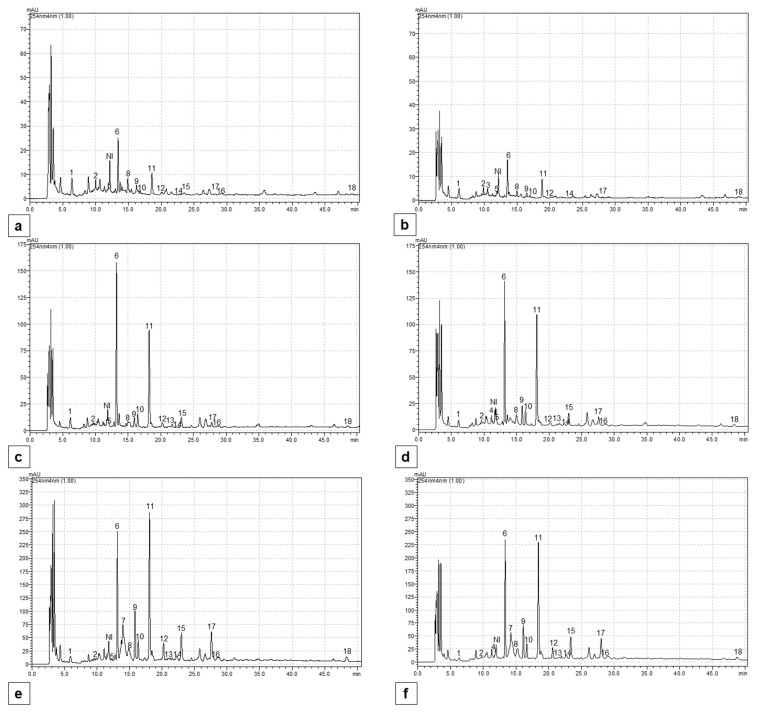
Chromatographic profile of the phenolic acids and flavonoids at 254 nm for different parts of the *Spergularia marina*: roots in slight (**a**) and strong (**b**) soil salinity, shoots in slight (**c**) and strong (**d**) soil salinity, inflorescence in slight (**e**) and strong (**f**) soil salinity. 1—3,4-dihydroxybenzoic acid; 2—catechin; 3—chlorogenic acid; 4—luteolin derivative; 5—epicatechin; 6–12—apigenin derivative; 13, 14—tricin derivative; 15—chicoric acid; 16—tricin derivative; 17—rosmarinic acid; 18—hesperetin; NI—not identified.

The content of flavonoids and phenolic acids in different parts of the studied plants *S. marina* and *G. maritima* at different levels of soil salinization was found (Table 2). Various parts of *S. marina* contained flavonoids such as catechin, hesperetin, epicatechin, apigenin derivative, luteolin derivative, and tricin derivative. Among the phenolic acids, protocatechuic acid and trace amounts of other derivatives of hydroxycinnamic acid (chlorogenic acid, chicoric acid, and rosmarinic acid) were found. It should be noted that with an increase in soil salinity, an increase in the content of hesperetin, epicatechin, and apigenin derivatives was found in the roots and shoots of *S. marina*, as well as luteolin derivatives in the shoots and inflorescences (Appendix A, Figure A1). In turn, with an increase in soil salinity in the roots of *S. marina*, the content of protocatechuic acid increased, whereas, in the shoots, it decreased.

**Figure A2:**
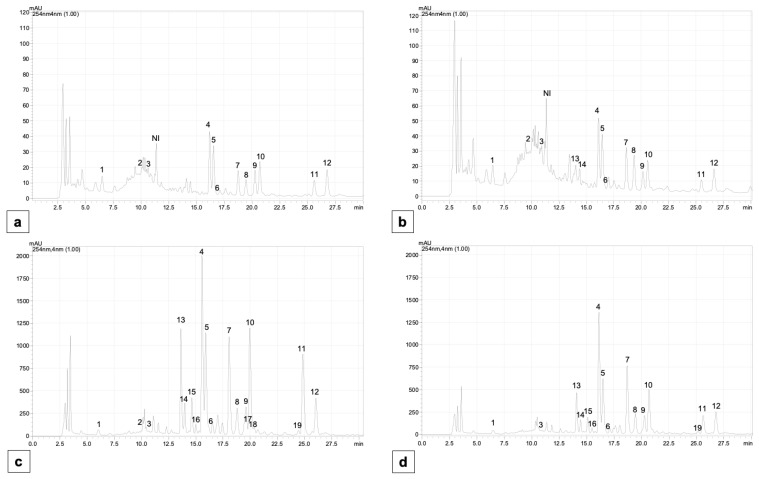
Chromatographic profile of the phenolic acids and flavonoids at 254 nm for different parts of the *Glaux maritima*: roots in slight (**a**) and strong (**b**) soil salinity, shoots in slight (**c**) and strong (**d**) soil salinity. 1—3,4-dihydroxybenzoic acid; 2—catechin; 3—chlorogenic acid; 4—quercetin derivative; 5—kaempferol derivative; 6—ferulic acid; 7, 8—kaempferol derivative; 9—quercetin derivative; 10—quercetin 3-β-d-glucoside; 11, 12, 13—quercetin derivative; 14—p-coumaric acid; 15, 16—quercetin derivative; 17—hyperoside; 18—quercetin 3-O-rutinoside; 19—kaempferol 3-O-glucoside; NI—not identified.

Flavonoids, such as hyperoside, isoquercitrin, astragalin, and trace amounts of catechin and rutin, as well as other derivatives of quercetin and kaempferol, were found in the shoots of *G. maritima*. Among the phenolic acids, protocatechuic, p-coumaric, ferulic, and traces of chlorogenic acids were detected. In general, for *G. maritima,* there was a tendency to reduce the content of flavonoids in roots and shoots with an increase in soil salinity (Appendix A, Figure A2).

### 2.6. Antioxidant Activity

In this study, the evaluation of antioxidant activity was conducted with assaying DPPH and ascorbic acid as a standard using a method related to the ability of antioxidants to bind radicals.

**Figure 4:**
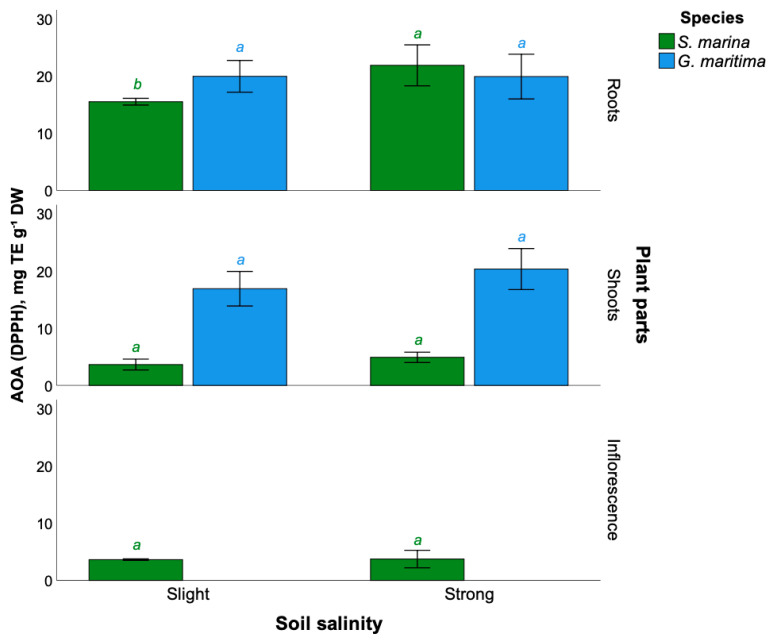
Antioxidant activity (DPPH) of extracts from different parts of the studied plants of *S. marina* and *G. maritima* at different levels of soil salinization. Different letters indicate significant differences among the different levels of soil salinity (*t*-test, *p* ≤ 0.05).

The extracts of *S. marina* roots had relatively high antioxidant activity under conditions of strong soil salinity (21.9 ± 3.6 mg TE g^−1^ DW), in comparison with a weak level of salinity (15.5 ± 0.6 mg TE g^−1^ DW). The antioxidant activity of stem and inflorescence extracts was 3–5 times lower compared to extracts from *S. marina* roots, and also did not depend on salinity conditions (Figure 4).

In turn, extracts of roots and shoots of *G. maritima* are characterized by high antioxidant activity. Extracts of plant shoots growing under conditions of strong soil salinization have slightly higher activity compared to a weak level of salinization (20.3 ± 3.6 and 16.9 ± 3.0 mg TE g^−1^ DW, respectively). At the same time, no statistically significant variation in antioxidant activity (*t*-test, *p* > 0.05), as measured by the DPPH method, was observed for roots and shoots in relation to the level of soil salinity.

### 2.7. Correlation between Phenolic Compounds Content and Antioxidant Activity

The diversity of compounds with different antioxidant mechanisms present in plants affects the antioxidant activity of their extracts. Antioxidant activity depends on the content of different classes of phytocomponents in the samples. Typically, phenolic compounds have a significant effect on the total antioxidant activity of extracts [50,51].

The correlation analysis conducted within the study showed a positive relationship between the content of different groups of phenolic compounds and the antioxidant activity of extracts of different parts of *S. marina* (Table 3). The correlation analysis confirmed a positive relationship between the antioxidant activity of *S. marina* root extracts and the total content of phenolic compounds (*r* = 0.909, *p* ≤ 0.01), protocatechuic acid (*r* = 0.859, *p* ≤ 0.01), and individual flavonoids (*r* = 0.754–0.893, *p* ≤ 0.05). However, the results relating to total flavonoid content (TFC) did not show significant correlations with both antioxidant activity and individual flavonoid content in all parts of *S. marina* studied.

The correlation analysis revealed a positive relationship between the antioxidant activity of *G. maritima* shoot extracts and the total content of phenolic compounds (*r* = 0.700, *p* ≤ 0.05), and the total content of hydroxycinnamic acids (*r* = 0.691, *p* ≤ 0.05). However, there is a negative correlation of AOA (DPPH) with the content of individual flavonoids—the derivatives of kaempferol (*r* = −0.681–−0.714, *p* ≤ 0.05) and protocatechuic acid (*r* = −0.678, *p* ≤ 0.05) (Table 4). The correlation analysis revealed no significant (*p* > 0.05) relationship between the content of different groups of phenolic compounds and the antioxidant activity of *G. maritima* root extracts.

### 2.8. Antibacterial and Antifungal Activity

The antibacterial and fungicidal activities of extracts from different parts of *S. marina* and *G. maritima*, which grow on soils with different levels of salinization, were studied with regard to the representative of Gram-negative bacteria *Escherichia coli*, the representative of Gram-positive bacteria *Bacillus subtilis*, and the saprotrophic yeast-like fungus *Candida albicans*. The results of the study are presented in Table 5. Taking into account the results presented in the table, it can be seen that the *S. marina* extracts did not show antibacterial activity. Only very weak fungicidal activity of shoots and inflorescences of *S. marina* was detected on soils with a high level of salinity.

Extracts from *G. maritima* shoots, however, exhibited some antibacterial effect, with minor activity against *E. coli;* the root extract, a 2 mg disk^−1^, hardly inhibited the colony growth of *B. subtilis*. Extracts from shoots and roots of *G. maritima* also exhibited a weak inhibitory effect on *Candida albicans*.

However, the standard antibiotic kanamycin (50 µg disk^−1^) that was used showed significant antibacterial and fungicidal activity against all the tested microorganisms (Table 5).

## 3. Discussion

### 3.1. Variation in Phenolic Compounds Content, Antioxidant, Antimicrobial, and Fungicidal Activity

There is now growing interest in halophytes due to their high bioactive content (primary and secondary metabolites), such as polyunsaturated fatty acids, vitamins, sterols, essential oils, polysaccharides, glycosides, and phenolic compounds. These biologically active substances possess potent antioxidant, antimicrobial, anti-inflammatory, and anti-tumor activity and, thereby, are key compounds for the prevention of various diseases [10]. Consequently, some halophytes have traditionally been used for medical and nutritional purposes. A promising area of research is the optimization of cultivation methods that maximize the yield of high-quality and functional plant products of wild plant populations and cultural species that are rich in target bioactive compounds, including the potential application of salinity as an effective method for increasing the content of secondary metabolites in plants, especially those of phenolic nature. From this standpoint, rare halophytic species of *S. marina* and *G. maritima*, which biological activity and secondary metabolites content has not been studied enough, are promising for use in the food and medical industries [23,24,25].

The results obtained in the present study supplement the knowledge about the qualitative composition of phenolic compounds in *S. marina* and the antioxidant activity of extracts. It was found that extracts of different parts of S. marina contained flavonoids (catechin, hesperetin, epicatechin, apigenin derivatives, luteolin derivatives, and tricin derivatives), as well as phenolic acids (protocatechuic acid, chlorogenic acid, chicoric acid, and rosmarinic acid).

In another study [52,53], phenyl lipid alkaloid and several phenolic compounds were identified from *S. marina*. These compounds were identified as 2,4-di-tert-butylphenol, N-hexacosanoylanthranilic acid, tryptophan, 4 hydroxybenzyol glucopyranoside, luteolin 6-C-β-D-glucopyranoside 8-C-β-D-(2-O-feruloyl)glucopyranoside, luteolin 6-C-β-D-(2-O-feruloyl)glucopyranoside 8-C-β-D-glucopyranoside, apigenin 6-C-β-D-glucopyranoside 8-C-β-D-(2-O-feruloyl)glucopyranoside, and apigenin 6-C-β-D-(2-O-feruloyl)glucopyranoside 8-C-β-D-glucopyranoside. The identified compounds can partially contribute to the antioxidant activity of *S. marina*. Thus, it was noted that compounds containing flavone 6,8-C-diglucoside and ferulic acid exhibited high antioxidant activity, as well as compounds containing luteolin and apigenin. Iranian researchers have shown [54] that *S. marina* contains relatively large amounts of phenols, flavonoids, tannins, and saponins in seeds, in the above-ground parts of the plant, and in the entire plant. The authors found that the number of phenolic compounds, flavonoids, tannins, and saponins was directly related to antioxidant activity. In another phytochemical study of *S. marina*, seven compounds were isolated from various plant extracts [31]. From the chloroform extract, β-sitosterol glycoside and tricin were isolated; in turn, dihydroferulic acid, vanillic acid, 4-hydroxybenzoic acid, uracil, and 8-hydroxy cuminoic acid were isolated from the ethyl acetate extract. Among the known compounds, in the authors’ opinion, tricin deserves special attention. Tricin is described to have specific biological properties, such as antiviral and anti-inflammatory action; antihistamine effects; and antioxidant, immunomodulatory, and anti-tuberculosis activities, which make this compound superior to other flavonoids and polyphenols in terms of agricultural, nutraceutical, and pharmaceutical value [55,56]. One of the most known features of tricin is its potential anti-tumor activity. However, the tricin content of *S. marina* is relatively low and is 0.398 mg/100 g wet weight [56].

Multiple drug resistance, including antibiotic resistance, has become a serious problem in pharmacotherapy as the number of infectious diseases caused by bacteria has increased. Special attention is paid to searching for new drugs that can be capable of solving this problem. Among the promising sources for searching for new medicinal compounds, one can identify secondary metabolites of plants belonging to different chemical classes [57].

In the present study, the antibacterial and fungicidal activity of extracts (10% DMSO) from different parts of *S. marina* against *Escherichia coli*, *Bacillus subtilis*, and *Candida albicans* was studied. Extracts showed no antibacterial activity, and weak fungicidal activity of extracts of shoots and inflorescences grown in soils with a high level of salinity was revealed against *C. albicans*. Similar results were obtained when studying the antimicrobial activity of the *S. marina* methanolic extract [54] with the disc diffusion method on eight bacterial species, including *Staphylococcus aureus*, *Escherichia coli*, *Bacillus subtilis*, *Pseudomonas aeruginosa*, *Streptococcus pneumoniae*, *Salmonella enterica*, *Klebsiella pneumoniae*, *Serratia marcescens,* and the fungus *Candida albicans*, where no inhibition zone was detected. In another study, the results of screening antibacterial and antifungal activity for different fractions showed [31] that all of the extracts and isolates of *S. marina* that were tested did not show antibacterial activity against Gram-negative *E. coli* and *P. aeruginosa*. However, the volatile fraction of *S. marina* petroleum ether extract had only antibacterial activity against the Gram-positive bacteria *Staphylococcus aureus*, *Micrococcus luteus*, and *Bacillus subtilis*. Extracts (petroleum ether, chloroform, ethyl acetate, n-butanol) were active against all the Gram-positive bacteria, with the exception of n-butanol, which was not active against *Staphylococcus aureus*. The only n-butanol extract was active against *Candida albicans*. The authors also tested the compounds isolated from the extracts (tricin, p-hydroxybenzoic acid, 8-hydroxy cuminoic acid) to compare their results with the activity of the extracts. Experiments have shown that the extracts were more active than the individual compounds. Tricin and p-hydroxybenzoic acid were active against *Bacillus subtilis*, *Micrococcus luteus,* and *Candida albicans*. In turn, 8-Hydroxy cuminoic acid was active only against *Candida albicans*. Thus, the available data allow us to conclude that various groups of phenolic compounds in *S. marina* extracts can be active against the Gram-positive bacteria studied in this work, as well as against the yeast-like fungus *Candida albicans*.

The study discussed in this article showed that *G. maritima* contains flavonoids and hydroxycinnamic acids, and the extracts of roots and shoots have high antioxidant activity, as well as weak antimicrobial activity against *Escherichia coli*, *Bacillus subtilis*, and fungicidal activity against *Candida albicans*. German researchers conducted screening tests the extracts *G. maritima* for the content of alkaloids, saponins, and phenolic compounds—namely, flavonoids and tannins [34]. The ethanolic extracts of *G. maritima* do not show the presence of saponins, flavonoids, and alkaloids. Ethanolic extracts of the above-ground parts of 28 plant species, including *G. maritima*, were screened by the same authors for activity against seven fungal, three yeast, five algae, and six bacterial species [35]. No activity was detected against all organisms tested for *G. maritima*. It is well known [58,59] that there are large differences in the chemical composition of different organs of the same plant. In addition, the chemical composition varies in plants from different geographical locations, climates, and soil conditions, which can explain the results obtained in this study and in the studies under discussion [34,35].

### 3.2. Effect of Soil Salinity on Phenolic Compounds Content

The present study showed an increase in TPC in the roots and shoots of *S. marina,* and in the shoots of *G. maritima,* in response to increased soil salinity. At the same time, the total content of flavonoids in all the studied parts of the two plant species remained unchanged. However, the content of individual flavonoids (hesperetin, epicatechin, apigenin derivatives, luteolin derivatives) in *S. marina* increased, for *G. maritima,* there was a tendency to reduce the content of flavonoids in roots and shoots with an increase in soil salinity. There was an increase in the total content of hydroxycinnamic acids in the roots of *Glaux maritima*, as well as an increase in the content of Protocatechuic acid in the roots and shoots of *Spergularia marina*. A positive relationship was established between the total content of phenolic compounds and the antioxidant activity of *S. marina* root extracts, as well as *G. maritima* shoots extracts.

In the present study, it is shown that the roots of *S. marina* and *G. maritima* are characterized by a high content of phenolic compounds and have a high antioxidant activity, which is most likely determined by their function. Plant roots use polyphenols to adapt to the rhizosphere, where communities of rhizospheric and root endospheric bacteria reside [60] and mycorrhizal symbiosis with fungi is formed [61,62], which is very important for the adaptation of halophytes to the coastal environment. For instance, flavonoids have beneficial impact on symbiosis, stimulating the spore germination, the development of hyphae, and the colonization of roots. Mycorrhiza increases the influx of assimilates to the roots [63].

Generally, halophytes are complex matrices containing a large variety of phenols [64]. However, the ability of plants to accumulate phenolic compounds in response to salinization depends on various factors, such as plant genotype, its ontogenetic state, organ-specific factors, and the intensity and duration of salinization. For example, treatment with 250 mM sodium chloride of *Aegiceras corniculatum* (L.) Blanco (a shrub that grows in moist soil in estuaries and river banks, often on the coastal edge of the mangrove zone) more than doubled the polyphenol content compared to control plants [65]. A study of salinity on the accumulation of biologically active compounds in the *Crithmum maritimum* L. halophyte revealed a significant accumulation of chlorogenic acid in leaves with increased salinity [66]. In another experiment [67], in a study of the effect of increased salinity on the total content of phenols, phenolic acids, antioxidant activity, and other compounds in *Brassica napus* var *oleifera* seedlings, it was shown that moderate salinization (25–50 mM NaCl) caused the greatest relative increase in the concentration of phenols. Salinization (50–100 mM NaCl) produced a markedly higher uptake of free DPPH forms from both early and late seedlings [67]. In turn, salinization did not have effects on the content of phenolic acids in seedlings. The authors identified a significant positive linear correlation between phenol content and antioxidant activity (*r* = 0.95) [67]. A study of *Cynara cardunculus* L. seedlings confirmed the importance of phenolic substances in the resistance of plants to stress conditions by showing that salt stress induced the synthesis of these molecules in proportion to the increase in the concentration of NaCl (0, 60, and 120 mM) [50]. As with total phenols, the trend of antioxidant activity (DPPH) was directly proportional to the salt concentration, demonstrating that the increase in polyphenols corresponds to greater antioxidant activity.

However, the accumulation of phenolic compounds in plants under salt stress is also associated with plant species, so in some plant species, phenolic compounds do not accumulate or vice versa, and a decrease in content is observed [68]. For example, the stress of salinity led to a decrease in the content of flavonoids and phenolic acids in broccoli leaves (*B. oleracea* var. *italica* of the Marathon variety), with flavonoid losses being higher [69]. A study of the effect of salinity on total phenol content in *Salvia macrosiphon* Boiss. showed a decrease in total phenol content in the leaves with an increase in the concentration of sodium chloride [70].

It can also be assumed that the accumulation of polyphenols in plants *S. marina* and *G. maritima* plays the role of protective metabolites in salinization. The phenolic composition of plants is one of the factors of plant adaptive variability in their adaptation to environmental conditions, including salinity. Salinization is known to cause oxidative stress due to the high production of reactive oxygen species (ROS), resulting in altered plant metabolism. Plants produce a large number of secondary metabolites to remove or detoxify ROS [68]. In response to salt stress, the phenylpropanoid biosynthesis pathway is stimulated, leading to the production of various phenolic compounds with strong antioxidant potential. Phenolic compounds help to neutralize harmful ROS in plants under salt stress [71]. Transcriptional regulation is an important tool to modulate flavonoid biosynthesis. Salt stress is associated with specific gene expression and synthesis of secondary metabolites to eliminate the negative effects of ROS [71,72]. For example, the *VvbHLH1* gene from grape has been shown to be involved in enhanced flavonoid production in transgenic *Arabidopsis thaliana* (L.) Heynh., regulating the biosynthesis pathway genes and conferring salt tolerance to plants [73]. In *Nicotiana tabacum* L., overexpression of *NtCHS1* gene plays an important role in flavonoid biosynthesis and in increasing salt tolerance [74]. In the halophytic plant *Aeluropus littoralis* (Gouan) Parl. expression of the *CWPRX* gene increased the content of phenolic acids in the leaf cell wall (ferulic acid, p-coumaric acid, and sinapic acid) [75].

## 4. Materials and Methods

### 4.1. Study Area and Plant Material

**Figure 5:**
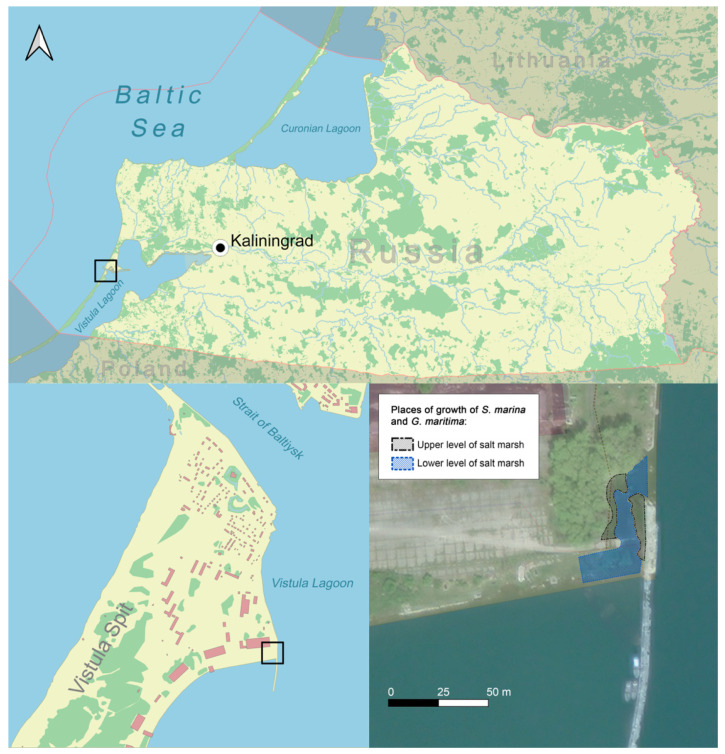
Map showing the growth sites of *S. marina* and *G. maritima*.

The collection of plants *S. marina* and *G. maritima* was carried out on 17 July 2021, on the Vistula Spit (Kaliningrad region), on the shore of the Vistula Gulf, on a periodically flooded meadow (salt marsh) characterized by herbaceous halophytic vegetation, where there grows, in addition to the studied species, *Trifolium fragiferum* L., *Triglochin maritima* L., *Tripolium pannonicum* (Jacq.) Dobrocz., and *Centaurium littorale* (Turner) Gilmour. Plants of *S. marina* favor disturbed areas and are often found on soil covered with water. *G. maritima* grows not only on the lower parts of the coast, together with species of the genus *Juncus* L., but also on the coastal bar as a dominant and codominant. The salt marsh is flooded with the waters of the bay (average salinity 3.5 psu) and more salty waters of the Baltic Sea (7.3 psu) during the period of surge winds (north and northwest direction) [76]. The water level, dropping during the storm periods, is on average about 0.5–0.7 m. On the investigated site, two levels can be conditionally distinguished, where the studied plant species grow [77]. The lower level is a shallow lowering of the topography, more often flooded, in which water accumulates after flooding (puddles form). The upper level of the salt marsh is somewhat elevated relative to the lower one, after flooding the water is not retained in these areas (Figure 5).

All experimental *S. marina* plants were harvested in the flowering phase and *G. maritima* plants in the vegetation phase. From each place of growth (lower and upper level of salt marsh), about 10–15 individuals of each species were collected from five test sites. The roots, shoots, and inflorescences of native plants of *S. marina* and only the roots and shoots of *G. maritima* were used as objects of study. In the laboratory, the plants were washed and dried at 60 °C to constant weight. The dried plants were milled to a particle size passing through a 1 mm mesh screen. Samples were placed in paper bags and stored in dark conditions at room temperature until further analysis.

### 4.2. Soil Analysis

Within each growing area of *S. marina* and *G. maritima*, soil samples were taken at a depth of 0–20 cm and combined into two samples for the upper and lower layers of the salt marsh, respectively. Soil samples were dried at room temperature, sieved through a 2 mm mesh sieve, and stored until further analysis.

A series of analyses including electric conductivity and pH in saturated aqueous soil extract (1:2.5) were performed to investigate soil salinity in the area under study, but soluble cations (Na^+^, K^+^, Ca^2+^) and anions (HCO_3_^−^, Cl^−^, SO_4_^2−^) were determined in a 1:5 water extraction. These analyses were performed using a multi-channel ionomer analyzer/conductometer ANION 7051. To determine the mass fraction of the dry residue, a gravimetric method was used based on the evaporation of the aliquot part of the filtered aqueous extract of the soil, and the drying of the resulting residue at a temperature of 105 °C followed by weighing [78].

### 4.3. Extract Preparation

The phenolic compounds were extracted with a 70% ethanol solution from crushed dry plant material of *S. marina* and *G. maritima*. A 0.2 g sample of plant material was ground in a mortar, placed in a 50 mL round bottom flask, and then poured into 30 mL of 70% ethanol. The flask was heated in a water bath and boiled for 30 min, then filtered through a paper filter into a volumetric flask. The extraction procedure was repeated three times. The resulting filtrates were combined, and the total volume was adjusted to 50 mL with a 70% ethanol solution.

### 4.4. The determination of Total Phenolic Content (TPC)

To determine the total content of phenolic compounds, the research team used the spectrophotometric method using the Folin–Ciocalteu reagent [59,79]. In total, 100 mL of sample, 300 μL of Folin–Ciocalteu reagent, 3 mL of sodium carbonate, and 3 mL of distilled water were added to 10 mL tubes and stirred. Distilled water was used as a control. After 20 min, the optical density of the solutions was determined at a wavelength of 720 nm on a UV-3600 spectrophotometer (Shimadzu, Japan). Gallic acid was used as the calibration standard. The total phenolic compound (TPC) content was estimated from the calibration curve and expressed in mg gallic acid equivalents per gram dry weight (Appendix B, Table A1).

### 4.5. The Determination of Total Flavonoid Content (TFC)

To determine the total content of flavonoids, a spectrophotometric method was used, based on the complexation reaction with aluminum chloride with the addition of a 10% acetic acid solution. In 10 mL tubes, 1 mL of extract, 2 mL of a 2% solution of aluminum chloride in 95% ethanol, and 1 drop of dilute acetic acid were added, and then the solution volume was adjusted to 10 mL with 95% ethanol. A reference solution was also prepared for each extract, namely, without the addition of a 2% aluminum chloride solution. After 30 min, the absorbance of the solutions was measured on a UV-3600 spectrophotometer (Shimadzu, Japan) at 410 nm [80]. Rutin was used as the calibration standard. TFC was expressed in mg of rutin equivalents per gram of dry weight (Appendix B, Table A1).

### 4.6. The Determination of Total Content of Hydroxycinnamic Acids (THA)

The research team used the spectrophotometric method using the Arno reagent to determine the total hydroxycinnamic acid content [51,81]. Then, 1 mLof vegetable extract, 2 mL of 0.5 M HCl, 2 mL of Arno reagent, and 2 mL of 8.5% NaOH were added to 10 mL tubes. The solution volume was adjusted to 10 mL with distilled water. For each extract, a reference solution was prepared separately without the addition of the Arno reagent. The absorbance of the solutions was measured on a UV-3600 spectrophotometer (Shimadzu, Japan) at 505 nm. Chlorogenic acid was used as the calibration standard. The total hydroxycinnamic acids (THA) content was estimated from the calibration curve and expressed in mg chlorogenic acid equivalents per gram dry weight (Appendix B, Table A1).

### 4.7. The Determination of Individual Phenolic Compounds

Individual phenolic compounds were determined by high-performance liquid chromatography with diode-array detection (HPLC-DAD) [51]. In preparation for HPLC, the extracts were filtered and dried. The resulting dry matter was then dissolved in a 10% methanol solution. The new extract was centrifuged (4500× *g*) for 15 min; the supernatant was filtered through a syringe filter (0.22 µm). The separation was performed on a Shimadzu LC-20 Prominence chromatograph with a Shimadzu SPD20MA diode array detector and an Agilent Zorbax Eclipse Plus C18 column (C18 250 × 4.6 mm^2^, 5 µm). The mobile phase included a solvent mixture of water/trifluoroacetic acid 99.9/0.1 (solvent A) and acetonitrile (B). Gradient mode was used for separation: 0 min—95% A, 5% B; 3 min—88% A, 12% B; 46 min—75% A, 25% B; 49.5 min—10% A, 90% B; 52 min—10% A, 90% B; 52.7 min—95% A, 5% B; 59 min—95% A, 5% B. We used a flow rate of 1.0 mL min^−1^, column temperature of 40 °C, and sample volume of 10 μL. Detection was carried out in the wavelength range of 180–900 nm.

Compounds of interest were identified by comparing their peak retention times and UV spectra to those of chromatographically pure samples. Chromatograms were processed using the LabSolutions software. Quantitative analysis of flavonoids was carried out according to calibration curves plotted in the concentration range of 5–100 μg/mL. The following Sigma-Aldrich standards (Sigma-Aldrich Rus, Moscow, Russia) were used: catechin, hyperoside, quercetin 3-o-rutinoside, quercetin 3-β-d-glucoside, kaempferol 3-o-glucoside, hesperetin, epicatechin, 3,4-dihydroxybenzoic acid, p-coumaric acid, ferulic acid, chlorogenic acid, chicoric acid, rosmarinic acid, apigenin 7-o-glucoside, apigenin 7-o-glucuronide, and luteolin 7-o-glucoside.

### 4.8. The Determination of Antioxidant Activity (AOA)

Antioxidant activity was determined by DPPH: 2.85 mL of DPPH solution was added to 100, 150, and 250 µL of extract. The volume was adjusted to 3.10 mL with ethyl alcohol. A mixture containing DPPH solution and 70% ethanol was used as the reference solution. The solutions were left for 60 min in a dark place. Absorbance reduction was measured at 515 nm on a UV-3600 spectrophotometer (Shimadzu, Japan) after 60 min of incubation of the reaction mixture at room temperature in the dark [82]. Ascorbic acid was used to build the calibration curve. Antioxidant activity was expressed in mg ascorbic acid equivalents per gram dry weight (Appendix B, Table A1).

### 4.9. Determination of Antimicrobial and Fungicidal Activity

The antibacterial and fungicidal activity of plant extracts was assessed by disc diffusion analysis [83]. The extracts of the plant species tested were filtered and dried, and the resulting dry matter was dissolved in a 10% Dimethyl sulfoxide (DMSO) solution. To study the antimicrobial activity of the extracts, a Gram-positive bacterium *Bacillus subtilis* (B-9865) and a Gram-negative *Escherichia coli* (DH5α) were selected, and a yeast-like fungus *Candida albicans* (Y-3108) was selected to study the fungicidal activity.

Sterile paper disks of filter paper with a diameter of 6 mm were impregnated with extracts in a concentration of 0.25, 0.5, 1.0, and 2.0 mg of extract per disk. Discs containing 50 µg of kanamycin were used as positive controls. Discs impregnated with 20 µL of 10% DMSO were used as a negative control.

The Mueller–Hinton medium was used for the analysis [84]. A 90 mm diameter petri dish was filled with 25 mL of culture medium. After solidifying the agar, 100 μL of a suspension of microorganisms, with a density of 0.5 McFarland, was inoculated onto the surface and then evenly distributed using a sterile Drigalsky spatula. The inoculated petri dishes were coated with sterile tweezers with extract disks and control disks and pressed for uniform contact with the medium. Petri dishes were incubated in a thermostat at 37 °C for 12 h. To establish antibacterial activity, the inhibition zone were measured in millimeters around the disc (diameter). The test was repeated three times to ensure reliability.

### 4.10. Statistical Analysis

IBM SPSS Statistics 23 was used for the statistical processing of the data obtained. To test statistical hypotheses and assess the significance of differences, the *t*-test and Mann–Whitney *U*-test were used. Statistical results are presented as mean ± standard deviation. The Pearson correlation coefficient was used to estimate the correlations of the quantitative features.

## 5. Conclusions

The present study showed that the studied halophytes *Spergularia marina* and *Glaux maritima* are characterized by a relatively high content of phenolic compounds, the total content of flavonoids, and high antioxidant activity, which is associated with the presence of phenolic compounds, individual flavonoids, and hydroxycinnamic acids in plants. The present results showed that the content of the studied substances was associated with soil salinization. Extracts of *S. marina* showed no antibacterial activity, while the weak fungicidal activity of extracts of shoots and inflorescences, grown on soils with a high level of salinity, was detected against *Candida albicans*. Extracts of roots and shoots of *G. maritima* have weak antimicrobial activity against *Escherichia coli* and *Bacillus subtilis,* and fungicidal activity against *Candida albicans*. Further experimental research is recommended to assess the effect of salinity on the content of phenolic compounds and the antioxidant activity of the studied halophytes. This includes the possible use of salinity as an effective method to increase the content of secondary metabolites in the studied plants for obtaining high-quality and functional plant products.

## Figures and Tables

**Table 1:** The results of the chemical analysis of soil of two habitats of the studied plant species.

Place of Growth	EC, dSm^−1^	pH	Dry Residue, %	Content of Soluble Cations and Anions, cmol_c_ kg^−1^					
				Cl^−^	Na^+^	K^+^	Ca^2+^	SO_4_^2−^	HCO^3−^
Lower level of salt marsh	1.38 ± 0.02 ^b^*	7.74 ± 0.02 ^a^	0.50 ± 0.05 ^b^	6.9 ± 0.2 ^b^	4.3 ± 0.1 ^b^	0.16 ± 0.01 ^b^	0.50 ± 0.04 ^b^	0.45 ± 0.08 ^b^	0.61 ± 0.06 ^a^
Upper level of salt marsh	4.02 ± 0.23 ^a^	7.56 ± 0.10 ^b^	1.42 ± 0.45 ^a^	16.5 ± 6.2 ^a^	9.1 ± 2.6 ^a^	0.25 ± 0.04 ^a^	3.00 ± 0.30 ^a^	0.67 ± 0.13 ^a^	0.64 ± 0.10 ^a^

* Different letters indicate significant differences between levels of soil salinity (Mann–Whitney *U*-test, *p* ≤ 0.05).

**Table 2:** Content of flavonoids and phenolic acids in different parts of the studied plants of *S. marina* and *G. maritima* at different levels of soil salinization.

	Content of Individual Phenolic Compounds, µg g^−1^									
	*S. marina*						*G. maritima*			
Plant Parts	Roots		Shoots		Inflorescence		Roots		Shoots	
Soil Salinity	Slight	Strong	Slight	Strong	Slight	Strong	Slight	Strong	Slight	Strong
Compounds	Flavonoids									
Catechin	190.1 ± 9.7 ^a^**	244.6 ± 47.4 ^a^	+ ***	+	+	+	+	+	+	-
Hyperoside	-	-	-	-	-	-	-	-	97.5 ± 10.9	-
Quercetin 3-O-rutinoside (rutin)	-	-	-	-	-	-	-	-	+	-
Quercetin 3-β-d-glucoside (isoquercitrin)	-	-	-	-	-	-	52.2 ± 9.4 ^a^	47.1 ± 5.6 ^a^	1711.0 ± 323.4 ^a^	587.5 ± 42.6 ^b^
Quercetin derivative *	-	-	-	-	-	-	273.9 ± 2.4 ^a^	165.4 ± 8.0 ^b^	5203.5 ± 1809.3 ^a^	2294.0 ± 240.4 ^b^
Kaempferol 3-O-glucoside (astragalin)	-	-	-	-	-	-	-	-	88.2 ± 22.9 ^a^	55.6 ± 6.0 ^b^
*Kaempferol derivative*	-	-	-	-	-	-	108.2 ± 14.4 ^b^	151.6 ± 17.0 ^a^	4248.0 ± 622.1 ^a^	2522.3 ± 329.2 ^b^
Hesperetin	32.9 ± 1.1 ^b^	51.9 ± 10.5 ^a^	48.9 ± 10.4 ^b^	71.9 ± 10.1 ^a^	111.0 ± 11.1 ^a^	100.6 ± 23.1 ^a^	-	-	-	-
Epicatechin	742.8 ± 42.4 ^b^	1012.6 ± 50.4 ^a^	+	77.1 ± 27.2	+	+	-	-	-	-
Tricin derivative	+	+	+	+	+	+	-	-	-	-
*Apigenin derivative*	311.3 ± 43.1 ^b^	410.7 ± 9.0 ^a^	1593.9 ± 327.6 ^b^	2170.9 ± 205.2 ^a^	2911.7 ± 176.9 ^a^	3541.4 ± 1340.4 ^a^	-	-	-	-
*Luteolin derivative*	-	-	-	102.9 ± 40.4	-	61.8 ± 19.9	-	-	-	-
	Phenolic acids									
3,4-Dihydroxybenzoic acid (protocatechuic acid)	35.9 ± 1.7 ^b^	52.4 ± 8.3 ^a^	31.4 ± 1.8 ^a^	24.6 ± 2.6 ^b^	27.1 ± 4.2 ^a^	21.4 ± 3.9 ^a^	21.7 ± 1.1 ^a^	19.6 ± 1.9 ^a^	46.8 ± 12.8 ^a^	38.6 ± 2.0 ^a^
p-Coumaric acid	-	-	-	-	-	-	-	-	16.9 ± 3.2 ^a^	21.7 ± 6.3 ^a^
Ferulic acid	-	-	-	-	-	-	+	+	+	16.1 ± 3.9
Chlorogenic acid	-	+	-	-	-	-	+	+	+	+
Chicoric acid	+	-	+	+	+	+	-	-	-	-
Rosmarinic acid	+	-	+	+	+	+	-	-	-	-

* The compounds identified by UV spectra and quantified by standard, with the same aglycon, are indicated in italics. ** Different letters indicate significant differences between levels of soil salinity (Mann–Whitney *U*-test, *p* ≤ 0.05). *** The sign “+” denotes trace amounts of compounds.

**Table 3:** Correlation matrix with the Pearson coefficient values for phenolic compounds and the antioxidant activity of *S. marina*.

				Roots				
Variables	AOA (DPPH) ^1^	TPC	TFC	Protocatechuic Acid	Hesperetin	Epicatechin	Apigenin Derivative	Catechin
AOA (DPPH)	1	0.909 **	−0.334 ^ns^	0.859 **	0.883 **	0.876 **	0.754 *	0.893 **
TPC		1	−0.189 ^ns^	0.797 **	0.837 **	0.870 **	0.800 **	0.753 *
TFC			1	−0.470 ^ns^	−0.418 ^ns^	−0.454 ^ns^	−0.403 ^ns^	−0.406 ^ns^
Protocatechuic acid				1	0.987 **	0.936 **	0.776 **	0.935 **
Hesperetin					1	0.909 **	0.757 *	0.954 **
Epicatechin						1	0.851 **	0.818 **
Apigenin derivative							1	0.663 *
Catechin								1
				Shoots				
AOA (DPPH)	1	0.492 ^ns^	0.050 ^ns^	−0.452 ^ns^	0.216 ^ns^	0.015 ^ns^	0.097 ^ns^	-
TPC		1	0.552 ^ns^	−0.846 **	0.720 *	−0.873 ^ns^	0.669 *	-
TFC			1	−0.450 ^ns^	0.672 *	−0.996 ^ns^	0.594 ^ns^	-
Protocatechuic acid				1	−0.768 **	0.978 ^ns^	−0.768 **	-
Hesperetin					1	−0.997 ^ns^	0.914 **	-
Epicatechin						1	−0.996 ^ns^	-
Apigenin derivative							1	-
				Inflorescence				
AOA (DPPH)	1	0.054 ^ns^	0.288 ^ns^	−0.558 ^ns^	−0.702 *	-	−0.082 ^ns^	-
TPC		1	0.064 ^ns^	−0.557 ^ns^	−0.346 ^ns^	-	0.208 ^ns^	-
TFC			1	0.281 ^ns^	0.099 ^ns^	-	−0.302 ^ns^	-
Protocatechuic acid				1	0.778 *	-	−0.517 ^ns^	-
Hesperetin					1	-	−0.122 ^ns^	-
Epicatechin						1	-	-
Apigenin derivative							1	-

^1^ AOA (DPPH), antioxidant activity determined by the DPPH (2,2-diphenyl-1-picrylhydrazyl) assay; TPC, total phenolic content; TFC, total flavonoid content; ** Correlation is significant at *p* ≤ 0.01; * correlation is significant at *p* ≤ 0.05; ^ns^, correlation is not significant (*p* > 0.05).

**Table 4:** Correlation matrix with the Pearson coefficient values for phenolic compounds and antioxidant activity of *G. maritima*.

					Roots					
Variables	AOA (DPPH) ^1^	TPC	TFC	THA	Isoquercitrin	Quercetin Derivative	Kaempferol Derivative	Astra Galin	Proto Catechuic Acid	p-Coumaric Acid
AOA (DPPH)	1	0.389 ^ns^	0.504 ^ns^	0.237 ^ns^	−0.389 ^ns^	0.025 ^ns^	0.310 ^ns^	-	−0.146 ^ns^	-
TPC		1	−0.127 ^ns^	0.419 ^ns^	−0.231 ^ns^	−0.237 ^ns^	0.397 ^ns^	-	−0.474 ^ns^	-
TFC			1	−0.255 ^ns^	−0.622 ^ns^	0.415 ^ns^	−0.350 ^ns^	-	−0.099 ^ns^	-
THA				1	−0.396 ^ns^	−0.889 **	0.791 **	-	−0.604 ^ns^	-
Isoquercitrin					1	0.289 ^ns^	−0.152 ^ns^	-	0.812 **	-
Quercetin derivative						1	−0.853 **	-	0.536 ^ns^	-
Kaempferol derivative							1	-	−0.348 ^ns^	-
Protocatechuic acid								-	1	-
					Shoots					
AOA (DPPH)	1	0.700 *	0.199 ^ns^	0.691 *	−0.521 ^ns^	−0.263 ^ns^	−0.681 *	−0.714 *	−0.678 *	−0.185 ^ns^
TPC		1	−0.201 ^ns^	0.486 ^ns^	−0.871 **	−0.607 ^ns^	−0.934 **	−0.842 **	−0.676 *	0.303 ^ns^
TFC			1	0.577 ^ns^	0.585 ^ns^	0.784 **	0.271 ^ns^	0.105 ^ns^	−0.270 ^ns^	−0.516 ^ns^
Isoquercitrin					1	0.904 **	0.903 **	0.839 **	0.495 ^ns^	−0.498 ^ns^
Quercetin derivative						1	0.668 *	0.657 *	0.136 ^ns^	−0.607 ^ns^
Kaempferol derivative							1	0.861 **	0.702 *	−0.391 ^ns^
Astragalin								1	0.768 **	−0.286 ^ns^
Protocatechuic acid									1	0.092 ^ns^
p-Coumaric acid										1

^1^ AOA (DPPH), antioxidant activity determined by the DPPH (2,2-diphenyl-1-picrylhydrazyl) assay; TPC, total phenolic content; TFC, total flavonoid content; THA, total hydroxycinnamic acids; ** Correlation is significant at *p* ≤ 0.01; * correlation is significant at *p* ≤ 0.05; ^ns^, correlation is not significant (*p* > 0.05).

**Table 5:** Antibacterial and fungicidal activity of extracts from different parts of the studied plants *S. marina* and *G. maritima* at different levels of soil salinization (the inhibition zones include the diameter of the disk—6 mm).

Art	Plant Parts	Soil Salinity	Diameter of Zone of Inhibition, mm					
			*E. coli*					
			Kanamycin	10% DMSO	Extract Concentration, mg Disk^−1^			
			50 µg Disk^−1^	20 µL Disk^−1^	0.25	0.50	1.00	2.00
*S. marina*	Roots	Slight	29.8 ± 0.2 ^a^*	-	-	-	-	-
		Strong	26.9 ± 1.0 ^b^	-	-	-	-	-
	Shoots	Slight	27.7 ± 2.9 ^a^	-	-	-	-	-
		Strong	29.0 ± 0.4 ^a^	-	-	-	-	-
	Inflorescence	Slight	29.3 ± 0.7 ^a^	-	-	-	-	-
		Strong	28.7 ± 0.7 ^b^	-	-	-	-	-
*G. maritima*	Roots	Slight	30.4 ± 1.9 ^a^	-	-	-	-	-
		Strong	29.2 ± 1.6 ^a^	-	-	-	-	-
	Shoots	Slight	29.4 ± 0.9 ^a^	-	-	-	-	-
		Strong	29.9 ± 2.5 ^a^	-	-	-	7.9 ± 0.4	9.5 ± 0.4
			*B. subtilis*					
*S. marina*	Roots	Slight	24.6 ± 1.7 ^a^	-	-	-	-	-
		Strong	24.6 ± 0.3 ^a^	-	-	-	-	-
	Shoots	Slight	26.5 ± 1.9 ^a^	-	-	-	-	-
		Strong	25.3 ± 0.2 ^a^	-	-	-	-	-
	Inflorescence	Slight	26.7 ± 0.6 ^a^	-	-	-	-	-
		Strong	26.0 ± 0.6 ^b^	-	-	-	-	-
*G. maritima*	Roots	Slight	26.1 ± 1.4 ^a^	-	-	-	-	7.0 ± 0.2
		Strong	26.0 ± 1.4 ^a^	-	-	-	-	-
	Shoots	Slight	24.8 ± 0.6 ^b^	-	-	-	-	-
		Strong	27.6 ± 0.7 ^a^	-	-	-	-	-
			*C. albicans*					
*S. marina*	Roots	Slight	27.8 ± 0.4 ^a^	-	-	-	-	-
		Strong	27.7 ± 0.6 ^a^	-	-	-	-	-
	Shoots	Slight	29.1 ± 2.0 ^a^	-	-	-	-	-
		Strong	29.3 ± 0.7 ^a^	-	-	-	6.4 ± 0.1	7.3 ± 0.8
	Inflorescence	Slight	28.4 ± 0.3 ^a^	-	-	-	-	-
		Strong	28.4 ± 1.1 ^a^	-	-	-	-	6.4 ± 0.1
*G. maritima*	Roots	Slight	27.8 ± 0.8 ^a^	-	-	-	6.8 ± 0.1 ^a^	7.5 ± 0.3 ^a^
		Strong	29.2 ± 3.2 ^a^	-	-	-	6.7 ± 0.1 ^a^	7.0 ± 0.1 ^b^
	Shoots	Slight	28.3 ± 0.7 ^a^	-	-	-	6.5 ± 0.1	6.5 ± 0.1 ^a^
		Strong	29.8 ± 1.0 ^a^	-	-	-	-	6.5 ± 0.1 ^a^

«-»—no inhibition zone was observed. * Different letters indicate significant differences between levels of soil salinity (Mann–Whitney *U*-test, *p* ≤ 0.05).

## Data Availability

The data presented in this study are available on request from the corresponding author.

## Appendix B

**Table A1:** Calibration curves of the equation of phenolic compound standards.

Method	Standard	Range, mg mL^−1^	Equation	R^2^
TPC	Gallic acid	0.02–0.30	y = 1.221x + 0.011	0.994
TFC	Rutin	0.025–0.500	y = 1.879x + 0.004	0.999
THA	Chlorogenic acid	0.01–0.30	y = 2.264x + 0.004	0.999
AOA (DPPH)	Ascorbic acid	0–0.34	y = −0.624x + 0.700	0.999
